# Interaction of Pd(II) and Pt(II) Amino Acid Complexes With Dinucleotides

**DOI:** 10.1155/MBD.1997.43

**Published:** 1997

**Authors:** Margarita Vicens, Amparo Caubet, Virtudes Moreno

**Affiliations:** Departament de Quimica Inorgànica Universitat de Barcelona Avinguda Diagonal 647 Barcelona 08028 Spain

## Abstract

The interaction of the dinucleotides d(ApG) and d(ApA) with [Pd(aa)Cl_2_], where aa = L- or D-histidine or the methyl ester of L-histidine, and with [Pt(Met)Cl_2_], where Met = L-methionine was studied by ^1^H and ^13^C NMR and CD measurements. In the case of the L-histidine and L-histidineOMe, the reaction with d(ApG) appeared to give the bifunctional adducts Pd(L-Histidine)N1(1)N7(2) and Pd(L-HisOMe)N1(1)N7(2), but the behavior with D-histidine suggested the formation of the monofunctional adduct Pd(D-His)N7(2). The reaction of L-histidine with d(ApA) seemed to form the bimetallic adduct (L-His)PdN7(1)N7(2)Pd(L-His). The Pt(II)-L-methionine complex in both reactions with d(ApG) and d(ApA) seemed to yield mainly adducts Pt(L-Met)N7(1)N7(2) but the existence of adducts Pt(L-Met)N1(1)N7(2) cannot be ruled out.


### INTERACTION OF Pd(II) and Pt(II) AMINO ACID COMPLEXES WITH DINUCLEOTIDES

Margarita Vicens, Amparo Caubet, and Virtudes Moreno*

Departament de Quimica Inorgànica, Universitat de Barcelona,
Avinguda Diagonal 647, 08028-Barcelona, Spain

**Abstract**

The interaction of the dinucleotides d(ApG) and d(ApA) with $[Pd(aa)Cl_2]$, where aa = L- or D-histidine or the methyl ester of L-histidine, and with $[Pt(Met)Cl_2]$, where Met = L-methionine was studied by $^1H$ and $^{13}C$ NMR and CD measurements. In the case of the L-histidine and L-histidineOMe, the reaction with d(ApG) appeared to give the bifunctional adducts Pd(L-Histidine)N1(1)N7(2) and Pd(L-HisOMe)N1(1)N7(2), but the behavior with D-histidine suggested the formation of the monofunctional adduct Pd(D-His)N7(2). The reaction of L-histidine with d(ApA) seemed to form the bimetallic adduct (L-His)PdN7(1)N7(2)Pd(L-His). The Pt(II)-L-methionine complex in both reactions with d(ApG) and d(ApA) seemed to yield mainly adducts Pt(L-Met)N7(1)N7(2) but the existence of adducts Pt(L-Met)N1(1)N7(2) cannot be ruled out.

**Introduction**

The mechanism of Pt(II) and other transition metal complexes binding to DNA has been widely studied in the last few years. This mechanism of action of antitumoral drugs is not completely understood, although the interaction of these compounds with DNA appears to be important. It has been interpreted as the formation of bidentate chelates with two N atoms from neighboring guanine bases(1). The best know compound in this class of substances is the classic cis-$PtCl_2(NH_3)_2$ discovered as an antitumor drug by Rosenberg several decades ago(2). Many authors have studied the reaction of possible new antitumoral complexes with nucleotides or oligonucleotides.

Hollis et al. (3) reported a new class of platinum antitumor agents: cis-$[PtCl(NH_3)_2(N\text{-het})]Cl$ (N-het is a heterocyclic amine like pyridine). Possible mechanisms of action of the new compounds, that must be different from that of the bifunctional cis-$Pt(NH_3)_2Cl_2$.have been proposed for this unusual new class of compounds. It has been suggested that the bifunctional active species could be generated *in vivo* ; another possible interaction of this compound could be monofunctional binding to guanines of the DNA, with N-het acting as an intercalator.

Reedijk and al. (4) studied the reaction of a new antitumoral complex of Pt(II), cis-$[PtCl(NH_3)_2$(4-methylpyridine)]Cl with the dinucleotide d(GpG) and they report the preference for platinum binding at the N7,N7 in the dinucleotide. In another paper, the same authors(5) studied the conformation of the Pt(en)-dinucleotides in relation to the $Pt(NH_3)_2$-dinucleotides by $^1H$ NMR and CD spectroscopy and they found only small differences in the spectra, suggesting the same structure for both adducts. Several authors have concluded that binding occurs by the formation of a bifunctional adduct by the N7 atoms of two neighboring guanine bases(6-8). Hydrogen bonding plays an important role in the stabilization of this pGpG-N7,N7 adduct. The preference for the guanine N7 could be related to at least two factors: 1) the influence of the 5'-phosphate and guanine O6, both of which can form hydrogen bonds to the amine group, and 2) the nature of the neighboring bases to this sequence(9-2). The influence of a 5'phosphate group on the reactivity of the N7 of a guanine in the reaction with platinum complexes was discussed.

A study of the guanine-O6 methylation of d(GpG) described the reduction of reactivity of the dinucleotide towards Pt(II) complexes(13) and the influence of the hydrogen bonding on the preference of binding to the guanine-N7.

The aim of our work is to study the nucleotide coordination sites and the possibility of chelate formation between the $[Pd(L\text{-Histidine})Cl_2]$, $[Pd(D\text{-Histidine})Cl_2]$, $[Pd(L\text{-HistidineOMe})Cl_2]$, $[Pt(L\text{-Methionine})Cl_2]$, and the d(ApG) and d(ApA) dinucleotides. The structures of the $[Pt(L\text{-Methionine})Cl_2]$, $[Pt(L\text{-Histidine})Cl_2]$, and $[Pd(L\text{-HistidineOMe})Cl_2]$, have been determined crystallographically(14, 15).

Several studies have been carried out on the synthesis of Pd(II) complexes analogues of Pt(II) anticancer drugs and their interaction with dinucleotides or other DNA components. It is apparent that the interest in the use of the Pd(II) complexes as kinetic models is now increasing (16-20).

### Materials and Methods

The palladium complexes and the platinum compound were synthesized and recrystallized following established procedures(14,15) using $K_2PdCl_4$ or $K_2PtCl_4$ (Johnson Matthey products) and the appropriate amino acid. The identity of the Pd/Pt derivatives was determined by infrared spectroscopy and elemental analysis. The deoxynucleotides sodium salts d(ApG) and d(ApA) were obtained from Sigma Chemicals and used without further purification. pH was adjusted by addition of DCl or NaOD solutions. Values of pH (hereafter denoted pH*) were not corrected for deuterium isotope effects. All the reactions, between 2-3 mmolar2-3 d(ApG) and d(ApA) were carried out in a sterilized NMR tube in $D_2O$ pH*7 at room temperature and were followed by $^1H$ NMR spectroscopy in function of time. Pd/Pt complexes in a 1:1 molar ratio were used. The $^1H$ NMR and $^{13}C$ NMR spectra were recorded with a Varian 300 MHz spectrometer. $D_2O$ was used as a solvent and TMS as internal reference. The CD spectra were recorded at room temperature on a Jasco 700 instrument from solutions in the same conditions ( using $H_2O$ pH 7 as solvent) and concentrations that studied by NMR at final time considered for the formation of the adducts. Electrospray mass Spectra of the mixtures prepared were obtained on a VG-QUATTRO, Fisons Instruments, at 80 ºC in dry $N_2$, using $MeOH/H_2O$ (1:1) as eluent with a flux of $7mL.min^{-1}$.

### Results and Discussion

The reaction between Pd(II) histidine complexes and the dinucleotides was carried out in $D_2O$ in a NMR tube and studied by $^1H$ NMR spectroscopy. The pH of the all solutions, in this case, was monitored to 6.8-7.2.

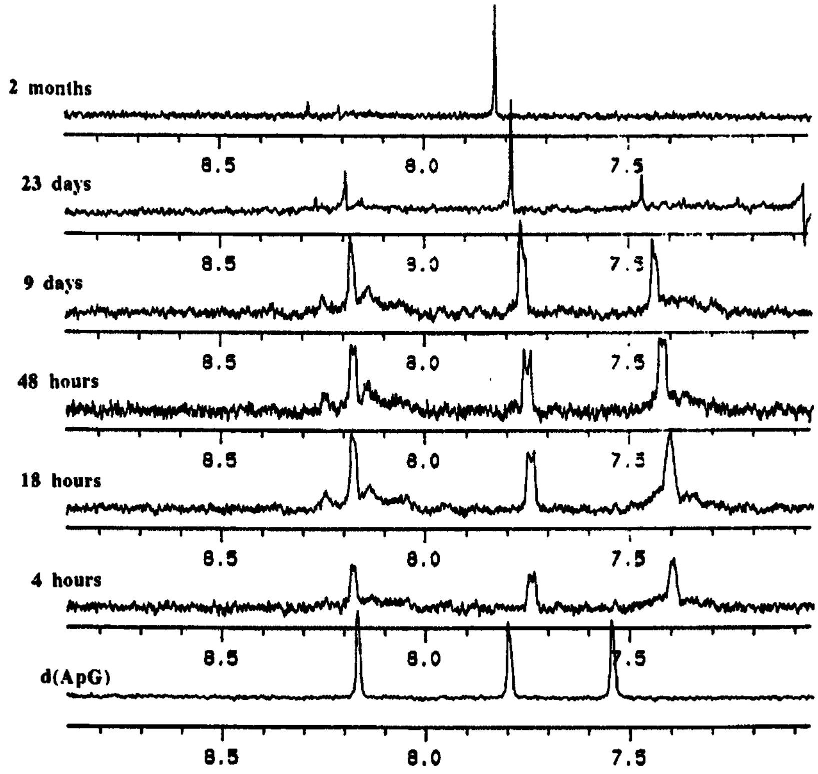

**Figure 1.** Aromatic proton zone (ppm) of $^1H$ NMR spectra of the reaction between d(ApG) and [Pd(L-His)$Cl_2$] over time.

The resonances at 8.17, 7.79, and 7.54 ppm in the spectrum of the free dinucleotide d(ApG) can be assigned to the H8 Ap, H2 Ap, and H8 pG respectively(21). The $^1H$ NMR spectra of the aromatic protons, for the d(ApG) and for a 1:1 mixture of d(ApG) and [Pd(L-His)$Cl_2$] over time are represented in Figure 1.

Addition of [Pd(L-His)$Cl_2$] produced upfield shifts of the signals corresponding to the H8 pG and to the H2 Ap while the H8 Ap remained unchanged. All the signals split in the following hours but after 23 days this splitting disappeared and the intensity of the signals corresponding to H8 Ap and H8 pG showed a progressive decrease until complete disappearance. At the same time, the pH* of the solution decreased from 7.2 at the beginning to 5.4. The Electrospray mass spectra of the final solution confirmed that the proton/deuterium exchange for H8 has occurred gradually, probably induced by the presence of the Pd(II) ion bound to the nearby N7. In contrast, this H/D exchange of the aromatic protons did not occur for the dinucleotide alone in solution in the same conditions. In the literature, several authors have shown that the exchange occurs when the metal ion is bound to the N7 site(22), which is considered as an additional aid in assigning the resonance or, spontaneously, in the case of alkaline solutions with pH*s above 9 and at 37 ºC or higher(23). In our case we have confirmed that pH* was always lower than 7.5 and decresed to ca. 5 over time and we have worked at 25 ºC.

In the spectrum corresponding to the reaction of the dinucleotide d(ApG) with [Pd(L-HisOMe)$Cl_2$] represented in Figure 2, no shifts in the signals were observed but splitting of the H2 Ap and H8 pG was detected. This splitting disappeared later. The H8 pG signal showed a slight decrease after two months. Four months later this signal remained with analogous intensity. The rate of the proton/deuterium exchange was smaller than in the case of the complex with L-histidine. The formation of the adduct may be a slow process due to the presence of the methyl group and binding of the Pd(II) to N7 may take a long time; as a consequence the induction of the exchange of the proton/deuterium may also be slower.

In the case of the reaction between [Pd(D-His)$Cl_2$] and d(ApG), see Figure 3, the H8 pG signal shifted upfield and the other two aromatic proton resonances did not move. Although the three signals split in the first steps of the reaction, the splitting disappeared over time. 45 days later, a decrease in the intensity of H8 Ap and H8 pG was observed. After three months, the behavior was similar to that of the L-histidine complex. The formation of the adduct was again more difficult than in the case of L-histidine, due now to its spatial configuration which is less suitable for the configuration of the dinucleotide[24]. Finally, the process of proton/deuterium exchange was likewise induced, and five months later, the signals had disappeared completely.

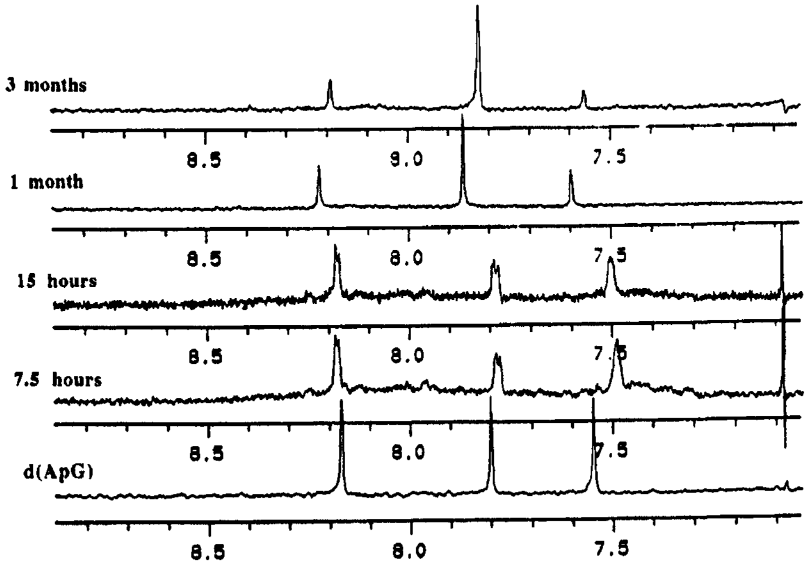

**Figure 2.** Aromatic proton zone (ppm) of $^1$H NMR spectra of the reaction between d(ApG) and [Pd(L-HisOMe)$Cl_2$] over time.

The spectrum of the dinucleotide d(ApA) exhibits two signals at 8.21 and 7.91 ppm that can be assigned to the H8 Ap and pA respectively [25-26]. The H2 Ap and H2 pA appear at 7.96 and 7.80 ppm respectively. The Figure 4 shows the aromatic resonances as well as the signal corresponding to the reaction of d(ApA) with [Pd(L-His)$Cl_2$] in function of time.

When the [Pd(L-His)$Cl_2$] is added a downfield shift in all the signals can be seen, but no split is observed in any case, even after 20 days. At this moment both resonances corresponding to the H8 Ap and pA decrease progressively and, simultaneously, a fall in pH and a shift of all the resonances in the spectrum can be detected. After four months, the signals corresponding to the H8 Ap and pA disappear like in the reaction with d(ApG). This indicates that the formation of the adduct with the adenine dinucleotide is slower than in the case of the adenin-guanine dinucleotide. However, it seems that in all cases the reaction evolves towards the formation of an adduct with the Pd(II) binding to N7, which induces the proton/deuterium exchange.

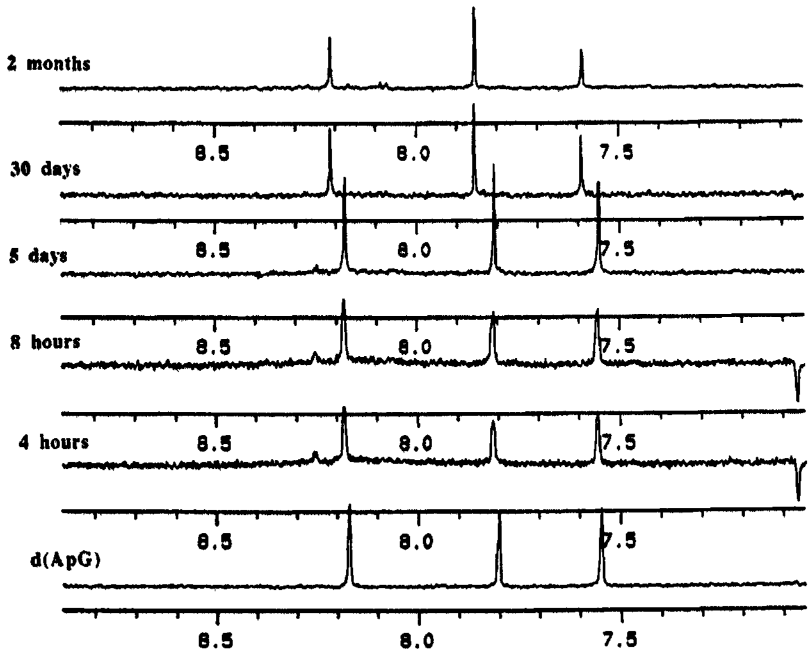

**Figure 3.** Aromatic proton zone (ppm) of $^1H$ NMR spectra of the reaction between d(ApG) and $[Pd(D\text{-}His)Cl_2]$ over time.

The reaction between the complex of L-methionine and the dinucleotides was carried out in $D_2O$ in an NMR tube and studied by $^1H$ and $^{13}C$ NMR spectroscopy. Previously, a pH-variation of the NMR signals for the nucleotides was carried out and the conditions suitable for the study were chosen. The pH* was monitored in all the cases at 3.

We can see in Table 1 the results for the $^1H$ NMR spectra for the two different Pt complex-dinucleotides.

**Table 1.** The most characteristic $^1H$ NMR chemical shifts (ppm) for d(ApA) and d(ApG) and their platinated complexes with $[Pt(L\text{-}Met)Cl_2]$.

| **d(ApA) assignment** | **d(ApA) (pH=3)** | **[Pt(L-Met)-d(ApA)] (pH=3)** |
|---|---|---|
| H2(Ap) | 7.73 | 8.17 |
| H8(Ap) | 7.86 | 8.19 |
| H2(pA) | 7.87 | 8.21 |
| H8(pA) | 8.15 | 8.37 |

| **d(ApG) assignment** | **d(ApG) (pH=3)** | **[Pt(L-Met)-d(ApG)] (pH=3)** |
|---|---|---|
| H2(Ap) | 7.60 | 8.10 |
| H8(Ap) | 7.96 | 8.25 |
| H8(pG) | 8.20 | 8.40 |

The results of the $^{13}C$ NMR study are shown in Table 2.

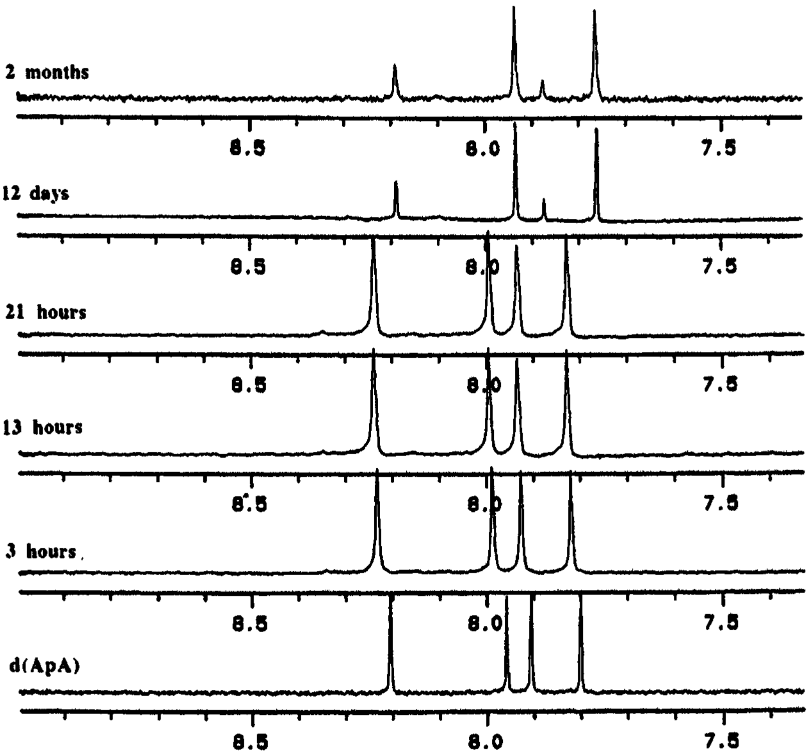

**Figure 4.** Aromatic proton zone (ppm) of $^1H$ NMR spectra of the reaction between d(ApA) and $[Pd(L\text{-}His)Cl_2]$ over time.

In the case of the d(ApA), the shiftings for the C8 of the two adenosine as well as the shiftings of the protons H8 indicate that the binding of the Pt-complex would be through the N7 giving a Pt(L-Met)-d(ApA)N7(1)N7(2) adduct. In the case of the dinucleotide d(ApG) the conclusion is, as in the first case, that the Pt complex could bind to the dinucleotide at the N7 positions, giving a Pt(L-Met)-d(ApG)N7(1)N7(2) adduct. However, the possibility of the presence of another complex Pt(L-Met)-d(ApG)N1(1)N7(2) cannot be ruled out[21].

**Table 2.** The most characteristic $^{13}C$ NMR chemical shifts (ppm) for d(ApA) and d(ApG) and their platinated complexes with $[Pt(L\text{-}Met)Cl_2]$.

| **d(ApA) assignment** | **d(ApA) (pH=3)** | **[Pt(L-Met)-d(ApA)] (pH=3)** |
|---|---|---|
| C8(pA) | 142.20 | 145.78 |
| C8(Ap) | 142.83 | 147.68 |
| C2(pA) | 154.44 | 155.9(br) |
| C2(Ap) | 154.70 | |
| **d(ApG) assignment** | **d(ApG) (pH=3)** | **[Pt(L-Met)-d(ApG)] (pH=3)** |
| C8(pG) | 140.30 | 144.80 |
| C8(Ap) | 142.20 | 147.80 |
| C2(pG) | 155.10 | 156.00 |
| C2(Ap) | 155.20 | 157.10 |

The behavior of the Pt(II) complexes with methionine is different from that of Pd(II) with histidine. In this case all the bands remain throughout the experiment because the presence of Pt(II), at pH 3, does not induce the proton/deuterium exchange and, moreover, the modifications in the position and forms of the signals are in agreement with the formation of the adducts by N7.

The instability of the Pt(II)-Histidine complexes and the polymeric character of some of the compounds isolated did not allow us to study their reactions with the dinucleotides. In the case of the $[Pd(L\text{-}Met)Cl_2]$ we observed that the reaction with the dinucleotide is fast and the signals corresponding to the H8 protons disappear immediately. This confirms that the presence of the Pd(II) ion in the neighborhood of N7 causes rapid proton/deuterium exchange. In the case of the Pt(II) derivatives no proton/deuterium exchange was observed at this low pH*.

A CD study of the solutions of the complexes of Pd(II) with L, D- Histidine and the methyl ester of L-Histidine gave complementary data which confirm the conclusions from the discussion based on the NMR study. The CD spectra of the solutions with the products of the reactions of d(ApG) and d(ApA) and the Pd(II) complexes are shown in Figures 5 and 6.

The spectrum of free d(ApG) presents two negative effects at 270 and 230 nm and one positive effect at 248 nm. In the cases of the reactions of d(ApG) with $[Pd(L\text{-}His)Cl_2]$ and $[Pd(L\text{-}HisOMe)Cl_2]$ a change in the sign of the band at 230 nm is observed which now appears in the adducts at 230 and 228 nm respectively, while no change is observed in the sign for the other bands (268 and 265 nm and 245 and 248 nm respectively). The ellipticity of the positive bands at 245 and 248 nm also changes. Theses modifications indicate that a conformational change takes place in relation to the free dinucleotide[23, 27]. In contrast, in the CD spectrum of the reaction mixture of d(ApG) with $[Pd(D\text{-}His)Cl_2]$ there is no change of sign, but only a considerable variation in the ellipticity of the band at 230 nm. Probably in this case the conformation of the adduct is the same as in the free dinucleotide.

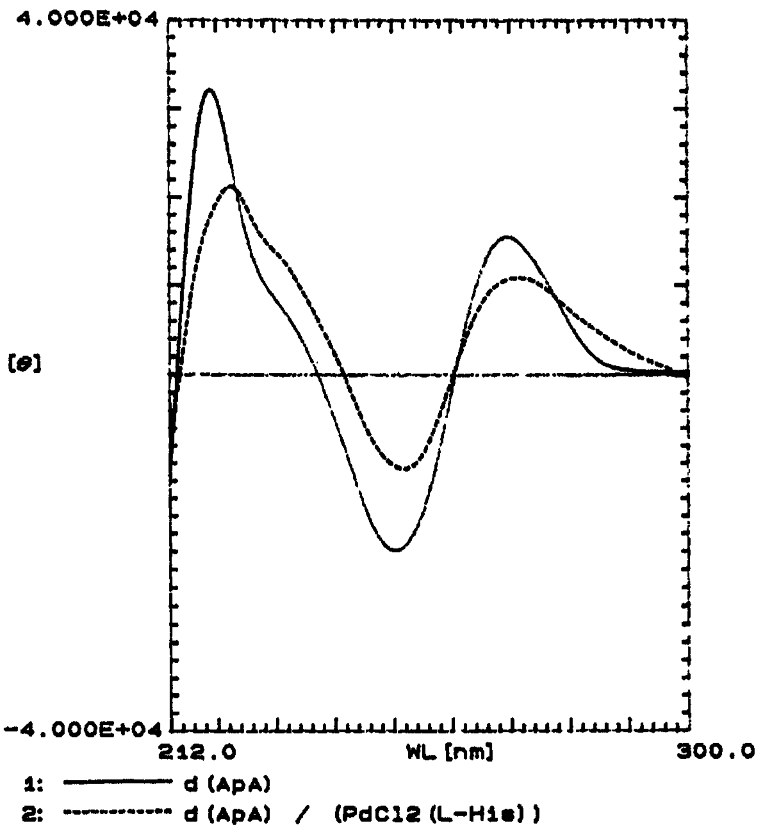

**Figure 5.** CD spectra of d(ApG) and the products of the reactions between d(ApG) and $[Pd(L\text{-}His)Cl_2]$, $[Pd(L\text{-}HisOMe)Cl_2]$, and $[Pd(D\text{-}His)Cl_2]$

The conformational changes for $[Pd(L\text{-}His)Cl_2]$ and $[Pd(L\text{-}HisOMe)Cl_2]$ agree with the formation of bifunctional adducts while the retention of the conformation in $[Pd(D\text{-}His)Cl_2]$ suggests that only monofunctional dinucleotide-Pd(II) adducts are present.

Finally, in the spectrum of free d(ApA) there are three bands, two positive at 270 and 218 nm, and one negative at 250 nm[27]. After reaction with $[Pd(L\text{-}His)Cl_2]$ no changes in the sign of any of the bands are observed. There are only slight modifications in the ellipticity indicating interaction with the Pd(II) complex. The retention of the conformation of the free d(ApA) can again be interpreted due to the presence of monofunctional adducts[25].

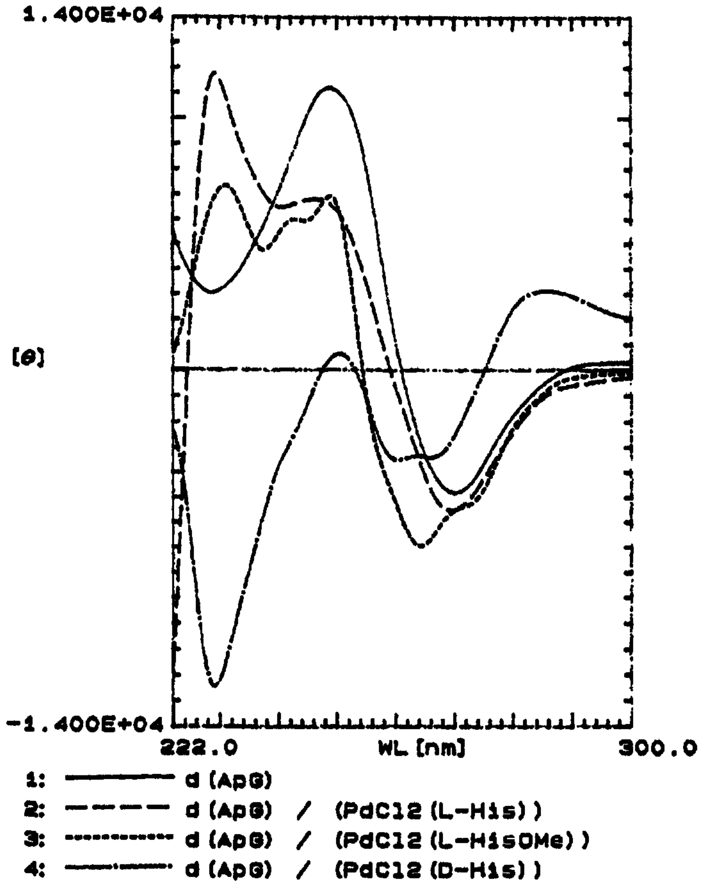

**Figure 6**. CD spectra of d(ApA) and the product of the reaction between d(ApA) and $[Pd(L\text{-}His)Cl_2]$

The CD study of the Pt(II)-methionine derivatives and the dinucleotides was not possible due to the presence of two isomers in solution. The sulfur atom of the methionine becomes chiral after binding to the metal ion and there are two possible stereoisomers in the adducts formed. It was not possible to separate them, and the CD spectra are those of the mixture of isomers.

### Conclusions

The CD and $^1H$ NMR study of the reaction of the dinucleotide d(ApG) with $[Pd(L\text{-}His)Cl_2]$ suggests the formation of the bifunctional adduct Pd(L-His)N1(1)N7(2) with an anti-anti conformation.

In the case of the reaction of the dinucleotide d(ApG) with $[Pd(L\text{-}HisOMe)Cl_2]$ the formation of the bifunctional adduct Pd(L-HisOMe)N1(1)N7(2) is also observed. The conformation is also anti-anti. The reaction is slower probably due to the presence of the methyl group.

In the reaction of the dinucleotide d(ApG) with $[Pd(D\text{-}His)Cl_2]$ only the monofunctional adduct Pd(D-His)N7(2) seems to be formed. The conformation is in this case anti-syn.

The CD and $^1H$ NMR study of the reaction of the dinucleotide d(ApA) with $[Pd(L\text{-}His)Cl_2]$ agrees with the formation of bimetallic adducts with formulae (L-His)PdN7(1)N7(2)Pd(L-His). The conformation in this case is also anti-syn.

The disappearance of H8 signals in some of the reactions over time indicates $^1H$ exchange with deuterium induced by the presence of the Pd(II) atoms binding to the N7.

The $^{1}H$ NMR and $^{13}C$ NMR study of the reaction of the dinucleotides d(ApG) and d(ApA) with $[Pt(L\text{-}Met)Cl_2]$ suggests the formation of the bifunctional adducts.

**Acknowledgements**
*We are grateful to DGICYT Ref. PB91-0806, to European Community HCM, ref. ERBCHRXCT 920016, to Ramon Guerrero for $^{1}H$ and $^{13}C$ NMR spectra of the methionine derivatives recorded and to Johnson Matthey for $K_2PdCl_4$ and $K_2PtCl_4$ supplied.*

**References**
1. B.K.Keppler (ed). *Metal Complexes in Cancer Chemotherapy.* VCH, Weinheim, 1993.
2. B.Rosenberg, L. VanCamp, J.E.Trosko, and V.H.Mansour, *Nature,* **222**, 385 (1969).
3. L.S.Hollis, A.R.Amundsen, and E.W.Stern, *J.Med.Chem.*, **32**, 128 (1989).
4. E.L.M.Lempers, M.J.Bloemink, J.Brouwer, Y.Kidani, and J.Reedijk, *J.Inorg.Biochem..* **40**, 23 (1990).
5. C.J. van Garderen, M.J.Bloemink, E.Richardson, and J.Reedijk, *J.Inorg.Biochem.*, **42,** 199 (1991).
6. A.M.J Fichtinger-Schepman, J.L. van der Veer, J.H.J den Hartog, P.H.M Lohman, and J. Reedijk, *Biochem.*, **24**, 707 (1985).
7. K.Inagaki, and Y.Kidani, *Inorg.Chim. Acta,* **106**, 187 (1985).
8. A.L.Pinto, and S.J.Lippard, *Biochim.Biophys.Acta,* **780**, 167 (1985).
9. S.E. Sherman, D.Gibson, A.H.J.Wang, and S.J. Lippard, *J.Am.Chem.Soc.*, **110,** 7368 (1988).
10. B.L.Heyl, K.Shinozuka, S.K.Miller, D.G.Vanderveer, and L.G. Marzilli, *Inorg.Chem.*, **24**, 661 (1985).
11. G. Admiraal, J.L van der Veer, R.A.G. de Graaf, J.H.J den Hartog, and J. Reedijk, *J. Am.Chem Soc.*, **109**, 592 (1987).
12. A. Loui, J. Kozelka, and J.C. Chottard, *Inorg. Chem.*, **27**, 2751 (1988).
13. A.F.Struik, C.T.M. Zuiderwijk, J.H. van Boom, L.I.Elding, and J.Reedijk, *J.Inorg.Biochem.*, **44**, 249 (1991).
14. A.Caubet, V.Moreno, E.Molins, and C.Miravitlles, *J.Inorg.Biochem.*, **48**, 135 (1992).
15. M.Calaf, A.Caubet, V.Moreno, M. Font-Bardia, and X.Solans, *J.Inorg. Biochem.*, **59**, 63 (1995)
16. M. Wienken, E. Zangrando, L. Randaccio, S. Menzer, and B. Lippert, *J. Chem. Soc. Dalton Trans.*, 3349 (1993).
17. S. Suvachittanont, H. Hohmann, R. van Eldik, and J. Reedijk, *Inorg. Chem.*, **32**, 4544 (1993).
18. K.J. Barnham, M.I. Djuran, U. Frey, M.A.Mazid, and P.J.Sadler, *J.Chem. Soc. Chem. Comm.*, **65** (1994).
19. T. Sugimuri, K. Shibakawa, H. Masuda, A. Odani, and O. Yamauchi. *Inorg. Chem.*, **32,** 4951 (1993).
20. C.Navarro-Ranninger, J.M. Pérez, F. Zamora, V.M. González, J.R. Masaguer, and C. Alonso, *J.Inorg. Biochem.*, **52**, 37 (1993).
21. F.J. Dijt, J.C. Chottard, J.P.Girault, and J. Reedijk, *Eur. J. Biochem.*, **179**, 333 (1989).
22. A. Eastman, M.M.Jennerwein, and D.L. Nagel, *Chem. Biol. Interactions,* **67**, 71 (1988).
23. B. van Hemelryck, J.P. Girault. G. Chottard, P. Valadon, A. Laoui, and J.C. Chottard, *Inorg. Chem.*, **26**, 787 (1987).
24. K.Inagaki, and Y. Kidani, *Inorg. Chem.*, **25**, 1 (1986).
25. J.C.Chottard, J.P.Girault, G.Chottard, J.Y.Lallemand, and D.Mansuy, *J.Am.Chem.Soc.* **102**, 5565 (1980).
26. C.H.Lee, F.S.Ezra, N.S.Kondo, R.H.Sarma, and S.S. Danyluk, *Biochem.*, **15**, 3627 (1976).
27. C.R.Cantor, M.M. Warshaw, and H. Shapiro, *Biopolymers,* **9**, 1059 (1970).

**Received: February 12, 1997 - Accepted: February 20, 1997 - Received in revised camera-ready format: February 21, 1997**

